# 应用SYBR green I荧光PCR研究*GSTM1*基因多态性与肺癌遗传易感性之间的相关性

**DOI:** 10.3779/j.issn.1009-3419.2010.05.23

**Published:** 2010-05-20

**Authors:** 德杰 郑, 峰 滑, 朝蓉 梅, 海粟 万, 清华 周

**Affiliations:** 300052 天津，天津医科大学总医院，天津市肺癌研究所，天津市肿瘤转移与微环境重点实验室 Tianjin Key Laboratory of Lung Cancer Metastasis and Tumor Microenvironment, Tianjin Lung Cancer Institute, Tianjin Medical University General Hospital, Tianjin 300052, China

**Keywords:** GSTM1 SYBR green I, 基因多态性, 肺肿瘤, GSTM1 SYBR green I, Genetic polymorphism, Lung neoplasms

## Abstract

**背景与目的:**

谷胱甘肽S转移酶M1（glutathione S-transferase M1, *GSTM1*）基因是参与体内多种致癌物代谢的重要的Ⅱ相代谢酶，其基因多态性被认为与人肺癌遗传易感性有关。本研究旨在探讨天津地区汉族人群*GSTM1*基因多态性与肺癌遗传易感性之间的关系。

**方法:**

采用SYBR green I实时荧光PCR熔解曲线分析方法检测天津地区265例肺癌患者和307例对照者*GSTM1*基因多态性，应用病例对照研究分析其与肺癌易感性及不同病理类型之间的关系。

**结果:**

① *GSTM1*（-）基因型在肺癌组和对照组的分布频率分别为56.6%和57.0%，两组之间无统计学差别（*χ*^2^=0.831, *P*=0.362）。经性别、年龄、吸烟状况调整后分析，携带*GSTM1*（-）基因型个体未增加患肺癌危险性（OR=0.840, 95%CI: 0.578-1.221, *P*=0.362）。②按病理分层分析*GSTM1*基因型与肺癌各病理类型之间的关系，其中鳞癌、腺癌、小细胞肺癌与其它病理类型肺癌患者*GSTM1*（-）基因型分布频率分别为65.8%、48.5%、47.8%和52.2%，与对照组相比，不同病理类型患者肺癌危险性均无明显统计学差异（*P*>0.05）。

**结论:**

在天津地区人群中*GSTM1*基因多态性与肺癌遗传易感性之间无相关性。

## Abstract (en)

**Background and objective:**

Glutathione S-transferase M1 (*GSTM1*) is an important phase Ⅱ metabolic enzyme gene which involves metabolism of various carcinogens in human body. Many studies showed that *GSTM1* genetic polymorphism was associated with lung cancer risk. The aim of this study is to investigate the relationship between *GSTM1* genetic polymorphism and lung cancer risk among Han nationality population in Tianjin district.

**Methods:**

*GSTM1* genetic polymorphism was detected by melting curve analysis of SYBR green I real-time PCR assay. Two hundred and sixty-fve histological confrmed lung cancer patients and 307 health controls were recruited in this case-control study and the relationship between *GSTM1* genetic polymorphism and lung cancer risk was investigated.

**Results:**

(1) The frequency of the *GSTM1*(-) in lung cancer and control groups was 56.6% and 57.0% respectively, and no signifcant difference was found between the distribution of the *GSTM1*(-) genotype in the two groups (*χ*^2^=0.831, *P*=0.362). (2) When considered the *GSTM1*(+) genotype as reference, there was no overall statistically increased lung cancer risk for carriers with the *GSTM1*(-) genotype adjusted by age, gender and smoking status (OR=0.840, 95%CI: 0.578-1.221, *P*=0.362). (3) The frequency of the *GSTM1*(-) genotype for squamous cell carcinoma, adenocarcinoma, SCLC and other histological types was 65.8%, 48.5%, 47.8% and 52.2% respectively, compared with the control group, no statistically increased lung cancer risk was observed (*P*>0.05).

**Conclusion:**

No evidence is found between *GSTM1* genetic polymorphism and lung cancer risk among Han nationality population in Tianjin district.

肺癌是严重危害人类健康的疾病，也是我国增长最快的恶性肿瘤^[1]^。流行病学研究^[2]^证实吸烟是导致肺癌的重要原因，但只有不到20%的吸烟者发生肺癌，同时肺癌的家族聚集现象提示个体的遗传易感性在肺癌发生中有重要作用。

谷胱甘肽S转移酶M1（glutathione S-transferase M1, GSTM1）是体内参与多种致癌物代谢的Ⅱ相代谢酶，研究^[3, 4]^发现GSTM1与肺癌的遗传易感性有关。SYBR green I染料实时荧光定量PCR技术是一种便捷、灵敏、特异和适用性广的基因分型、基因表达的检测方法，在基因鉴别、基因表达和基因分型实验中应用范围不断增多^[5]^。与传统PCR方法相比，该方法具有操作简便、污染少、高通量的特点。目前国内关于*GSTM1*基因多态性与肺癌关系的研究多采用普通PCR法，本实验采用SYBR green I染料法实时PCR结合熔解曲线分析方法研究天津地区人群*GSTM1*基因型分布，并研究*GSTM1*基因与肺癌遗传易感性之间的关系。

## 1 材料与方法

### 1.1 研究对象

本研究共纳入肺癌患者265例，为2008年3月-2009年7月天津医科大学总医院胸部肿瘤中心住院，并经外科手术和病理检查确诊的肺癌患者。男性病例190例，女性病例75例，平均年龄60岁；其中，鳞癌120例，腺癌99例，小细胞肺癌23例，其它（大细胞癌、腺鳞癌等）23例。307例对照人群为同期于天津医科大学总医院健康体检中心常规体检人群，男性218例，女性89例，平均年龄60岁，无呼吸系统疾病及肺癌家族史。本研究肺癌患者和对照人群一般资料见表 1。

**1:** 肺癌组和对照组一般资料 Demographic characteristics of lung cancer cases and controls

	Case (*n*, %)	Control (*n*, %)	*P*
Total	265 (100%)	307 (100%)	
Gender			
Male	190 (71.7%)	218 (71.0%)	0.834
Female	75 (28.3%)	89 (29.0%)	
Age			
Mean±SD	60.39±9.58	60.38±10.05	0.987
< 60	121 (45.6%)	144 (46.9%)	0.803
≥60	144 (54.4%)	163 (53.1%)	
Smoking status			
Never	86 (32.4%)	166 (54.1%)	< 0.001
< 30 pack-year	44 (16.6%)	99 (32.2%)	
≥30 pack-year	127 (47.9%)	41 (13.4%)	
Missing	8 (3.0%)	1 (0.3%)	
Histology			
Squamous cell	120 (45.3%)		
Adenocarcinoma	99 (37.3%)		
Small cell carcinoma	23 (8.7%)		
Others	23 (8.7%)		

### 1.2 流行病学调查

制定统一流行病学调查量表，内容包括：一般人口学特征、吸烟、饮酒、职业，以及既往疾病史、肿瘤及呼吸系统家族史等。吸烟者定义为每天至少吸一支烟，持续半年以上者。

### 1.3 样本采集

分别采集肺癌患者术前和对照组外周静脉血3 mL，EDTA抗凝，-80 ℃冰箱保存。

### 1.4 DNA提取

采用Axygen外周血DNA提取试剂盒（Axygen Biosciences公司）手工提取人基因组DNA，提取步骤按操作说明书进行，提取的DNA保存于-20 ℃冰箱。

### 1.5 *GSTM1*基因型检测和结果判断

#### 1.5.1 *GSTM1*基因型检测

*GSTM1*基因型检测方法采用SYBR green I实时荧光PCR方法。引物由赛百盛公司合成。GSTM1上游引物：5’-GAACTCCCTGAAAAGCTAAAGC-3’；下游引物：5’-GTTGGGCTCAAATATACGGTGG-3’。内对照β-globin上游引物：5’-CAACTTCATCCACGTTCACC-3’；下游引物：5’-GAAGAGCCAAGGACAGGTAC-3’。SYBR^®^R Premix Ex Taq^TM^试剂盒购自大连宝生物公司。反应体系：总体积为25 μL，其中模板DNA 2 μL（100 ng），GSTM1、β-globin每种上下游引物各0.5 μL，SYBR^®^R Premix Ex Taq^TM^(2X) 10 μL，ROX Reference Dye Ⅱ 0.5 μL，ddH_2_O 10.5 μL；以ddH_2_O替代模板作为阴性对照。反应在美国ABI公司7500 Real Time PCR仪上进行，反应条件按说明书两步法PCR标准扩增程序进行：95 ℃变性10 s；95 ℃、5 s，60 ℃、34 s，40个循环；然后进入熔解曲线分析阶段。

#### 1.5.2 基因型判断

根据PCR产物熔解曲线分析判断*GSTM1*基因的基因型。熔解曲线中分别在82.2 ℃±0.2 ℃和86.0 ℃±0.2 ℃分别出现两个峰判断为*GSTM1*（+）基因型，只在86.0 ℃±0.2 ℃出现一个峰判断为GSTM1（-）基因型。选取10%实时荧光PCR扩增产物在1.5%琼脂糖凝胶上进行电泳，比较其与熔解曲线的一致性。

### 1.6 统计学方法

采用SPSS 16.0软件对数据进行统计学分析。配对*t*检验比较病例组和对照组间性别、年龄；*χ*^2^检验比较两组间吸烟情况及不同基因型在两组间的频率分布；非条件*Logistic*回归分析计算OR和95%CI以估计不同基因型与肺癌风险之间的相关性，以*P* < 0.05为差异具有统计学意义。

## 2 结果

### 2.1 患者基本资料统计结果

肺癌组和对照组年龄和性别比例无统计学差异，肺癌组吸烟者比例高于对照组（67.2%和45.9%），差异具有统计学意义（*χ*^2^=86.87, *P* < 0.05）。

### 2.2 熔解曲线特异性分析

本研究在熔解曲线分析的基础上进一步进行实时荧光PCR产物的电泳分析，结果显示该实验具有优良的特异性。SYBR green I实时荧光PCR熔解曲线分析检测*GSTM1*基因分析结果见图 1，实时荧光PCR扩增产物凝胶电泳结果见图 2。

**1:**
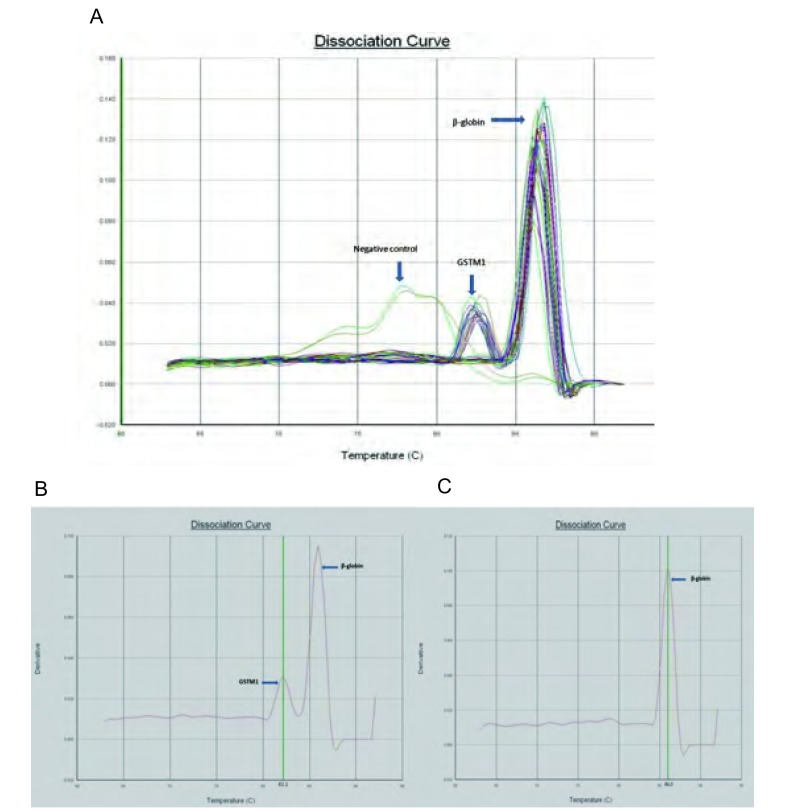
SYBR greenⅠ实时荧光PCR方法检测GSTM1熔解曲线峰图 Realtime PCR assay with SYBR green I and melting curve analysis of *GSTM1* gene. A: Differentiation of GSTM1, *β*-globin and negative controls by performing melting curve analysis; B: The present genotype of *GSTM1* gene gives one peak with an average melting point of 86.0 ℃ (*β*-globin) and another with an average melting point of 82.2 ℃ (GSTM1); C: the null genotype of *GSTM1* gene only gives a single melting point at 86.0 ℃ (*β*-globin).

**2:**
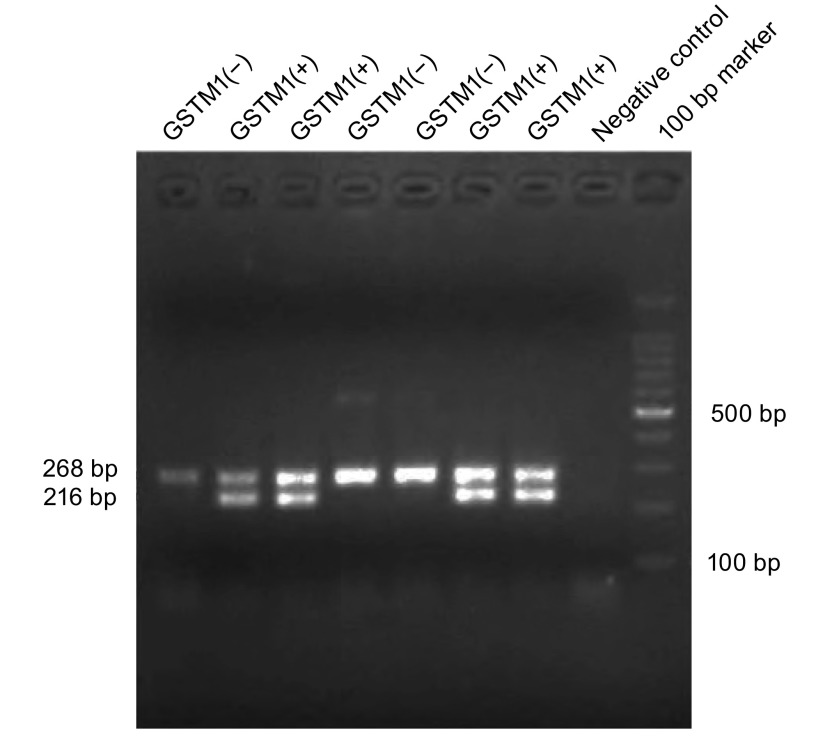
SYBR green I实时荧光PCR方法检测GSTM1扩增产物电泳图 Agarose gel electrophoresis graph of the SYBR green I Realtime PCR assay products

### 2.3 *GSTM1*基因型分布及其与肺癌风险性的关系

本研究中肺癌组与对照组*GSTM1*基因型分布频率无明显差异，*GSTM1*（-）基因型在肺癌组和对照组中的分布频率分别为56.6%和57.0%，差异无统计学意义（*χ*^2^=0.831, *P*=0.362）。以携带*GSTM1*（+）基因型的个体为参照，携带*GSTM1*（-）的个体患肺癌的风险性未见增加（OR=0.840, 95%CI: 0.578-1.221, *P*=0.362）（表 2）。

**2:** 肺癌组和对照组*GSTM1*基因型分布及与肺癌易感性之间的关系 Distribution of *GSTM1* genotype and association with lung cancer risk

Genotype	Group	
	Control (*n*, %)	Lung cancer (*n*, %)
GSTM1(-)	175 (57.0%)	150 (56.6%)
GSTM1(+)	132 (43.0%)	115 (43.4%)
Total	307 (100%)	265 (100%)
There was no statistically significant difference in the distribution of the GSTM1(-) genotype among cases and controls (*χ*^2^=0.831, *P*=0.362). Compared with the GSTM1(+) carriers, no increased lung cancer risk was observed for carriers with the GSTM1(-) genotype (OR=0.840, 95%CI: 0.578-1.221, *P*=0.362).		

### 2.4 *GSTM1*基因型与肺癌病理类型之间的关系

*GSTM1*基因型与肺癌病理类型之间的关系见表 3。120例鳞癌、99例腺癌、23例小细胞肺癌及23例其它类型肺癌中*GSTM1*（-）基因型所占比例分别为65.8%、48.5%、47.8%和52.2%，各组与对照组*GSTM1*（-）基因型百分比（57.0%）相比差别无统计学意义（*P* > 0.05）。各不同病理类型肺癌发生肺癌的危险性与对照组相比无差别（*P* > 0.05）。

**3:** *GSTM1*基因型与肺癌病理类型之间的关系 Association between *GSTM1* genotype and lung cancer histological types

Group	Genotype		OR^a^	95%CI	*P*^a^
	GSTM1(-) (*n*, %)	GSTM1(+) (*n*, %)			
Control	175 (57.0%)	132 (43.0%)	1		
SC	79 (65.8%)	41 (34.2%)	0.763	0.445-1.306	0.324
AC	48 (48.5%)	51 (51.5%)	1.472	0.908-2.388	0.117
SCLC	11 (47.8%)	12 (52.2%)	1.355	0.559-3.285	0.501
Others	12 (52.2%)	11 (47.8%)	1.321	0.543-3.211	0.539
^a^: OR and *P* value adjusted by age, gender and smoking status.					

## 3 讨论

大规模流行病学研究发现肺癌受吸烟、环境及遗传等多因素的共同影响。*GSTM1*基因是GSTs家族的一员，其编码的GST-μ蛋白能催化体内PAH类化合物失活而失去致突变性。*GSTM1*基因缺失者因无编码蛋白产生而导致对特定致癌物解毒能力下降，因此*GSTM1*（-）基因型可能与肿瘤发生有关^[6]^。

SYBR green I实时荧光PCR方广泛检测DNA的相对定量和绝对定量试验，具有迅速、灵敏、特异、能实现高通量的特点。Tiwawech等^[7]^建立了SYBR green I时荧光PCR结合熔解曲线分析检测泰国人群鼻咽癌患者*GSTM1*基因多态性的方法，与传统PCR方法分型相比结果完全一致。国内李一荣等^[8]^应用该方法检测120例HLA-B27已知基因型的标本并与传统PCR方法比较，二者分型结果完全一致。本实验中亦采用SYBR green I时荧光PCR结合熔解曲线分析方法研究天津地区汉族人群*GSTM1*基因多态，并用琼脂糖凝胶电泳进一步验证荧光PCR反应结果，结果显示该方法具有优良的特异性和敏感性。本研究中肺癌组和对照组*GSTM1*（-）基因型频率分别为56.6%和57.0%，与国内文献报道^[9, 10]^结果相一致。

关于*GSTM1*（-）与肺癌风险性的关系研究^[11, 12]^，结论并不一致。Benhamou等^[13]^进行的一项总人数超过18 000例、包含高加索人群和亚洲人群的*meta*分析结果表明单一*GSTM1*（-）基因型并不增加肺癌风险（OR=1.08, 95%CI: 0.98-1.18）。国内苏艳华等^[14]^入选8篇文献的*meta*分析认为*GSTM1*（-）基因型增加患肺癌的风险性（OR=1.65, 95%CI: 1.26-2.15）。钱碧芸等^[15]^进行一项天津市居民病例对照研究分析了108例肺癌患者和108例健康对照者*GSTM1*基因多态与肺癌易感性之间的关系，结果显示*GSTM1*（-）基因型携带者肺癌风险增高，差异具有统计学意义（OR=1.84, 95%CI: 1.03-3.29, *P* < 0.05），而曾勉等^[16]^对广东籍的91例肺癌患者和91例对照的研究显示*GSTM1*（-）基因型携带者并不增加患肺癌危险（OR=1.26, 95%CI: 0.69-2.30）。本实验中研究了天津地区265例肺癌患者和307例健康体检者，结果显示*GSTM1*（-）基因型不增加肺癌风险性（OR=0.840, 95%CI: 0.578-1.221, *P*=0.362），出现不同结果可能与样本量、不同地区人群差异等因素有关。

一项包括1 971例肺癌病例和2 130例的关于亚洲人群的的汇总分析^[17]^结果显示，*GSTM1*（-）基因型增加了患肺鳞癌的风险（OR=1.36, 95%CI: 1.05-1.77）。本实验中，与*GSTM1*（-）基因型与肺癌关系总体结果相一致，分层分析*GSTM1*（-）基因型的各型肺癌患者患癌危险性均未见增加。

恶性肿瘤的发生是受多因素长期影响的发展阶段，单一基因发生变化在起恶性肿瘤的发生中作用有限，我们的研究结果显示单一*GSTM1*（-）基因改变与肺癌危险性无关，需要进一步大样本量验证及研究*GSTM1*与其它基因的之间的相互作用。
